# The Persistence of Influenza Infection

**DOI:** 10.3201/eid1611.100974

**Published:** 2010-11

**Authors:** Julian W. Tang

**Affiliations:** Author affiliation: National University Hospital, Singapore

**Keywords:** Influenza, viruses, persistence, shedding, immunocompromised, stool, respiratory secretions, letter

**To the Editor:** The report by Pinsky et al. (*1*) is interesting, but it raises some major questions. The finding of influenza virus in stool is not new (*2*). Of more interest is their statement regarding the prolonged shedding of influenza virus in the stool (for >2 months) and respiratory secretions (for >1.5 years). How frequently were respiratory samples collected and tested to confirm that the same virus was shed for these periods in these samples? Influenza virus, like most other acute respiratory viruses, typically does not cause long-term latent or persistent infections in humans. The authors need to exclude the possibility of frequent reinfection with contemporary circulating seasonal hemagglutinin 1 (H1) influenza viruses. However, they do not provide any data to this effect.

Currently, with the wider availability and more stringent expectations of modern molecular techniques, such data might be obtained by collecting and sequencing several genes (ideally full genomes) from contemporary circulating seasonal H1 viruses and comparing them, phylogenetically, with the virus shed, contemporaneously, from the child, at monthly intervals, for example (if the child tolerates this testing). Even with this testing frequency, several influenza infection episodes may go undetected. Although the child’s virus, if it truly persists, may undergo some minor host-induced mutations, new infections with seasonal H1 viruses will likely demonstrate a greater, sudden sequence variability, which enables them to be relatively easily distinguished from the more minor, gradually accumulated mutations that can be seen in a persisting infection (*3*).

Second, ribavirin is not recommended for treating influenza infection (*4**,**5*). Can the authors explain why this child was taking ribavirin for influenza infection, and how often was his condition treated with this drug during the 1.5 years when influenza H1 was shed? Was his treatment regimen eventually changed? Currently, the recommended treatment for influenza is with the neuraminidase inhibitors (oseltamivir, zanamivir), which have a much safer adverse effect profile, and their effectiveness has been shown to be cost-effective (*5*).

## References

[1] Pinsky BA, Mix S, Rowe J, Ikemoto S, Baron EJ. Long-term shedding of influenza a virus in stool of an immunocompromised child. Emerg Infect Dis. 2010;16:1165–7. 10.3201/eid1607.091248 20587197 PMC3321893

[2] Wootton SH, Scheifele DW, Mak A, Petric M, Skowronski DM. Pediatr Infect Dis J. 2006;25:1194–5.Detection of human influenza virus in the stool of children. 10.1097/01.inf.0000245097.95543.11 17133173

[3] Baz M, Abed Y, McDonald J, Boivin G. Clin Infect Dis. 2006;43:1555–61.Characterization of multidrug-resistant influenza A/H3N2 viruses shed during 1 year by an immunocompromised child. 10.1086/508777 17109288

[4] Hayden FG, Pavia AT. Antiviral management of seasonal and pandemic influenza. J Infect Dis. 2006;194(Suppl 2):S119–26. 10.1086/507552 17163384

[5] Whitley RJ. Expert Opin Drug Metab Toxicol. 2007;3:755–67.The role of oseltamivir in the treatment and prevention of influenza in children. 10.1517/17425255.3.5.755 17916060

