# Supplementary material for: The Persistence of Influenza Infection
Source: Emerg Infect Dis. 2010 Nov;16(11):1817–9. doi: 10.3201/eid1611.100974 (PMC3294535; doi:10.3201/eid1611.100974)
Supplement: Related texts

# Reply

**Authors:** Benjamin Pinsky, Ellen Jo Baron

**Affiliations:** Author affiliations: Stanford University School of Medicine, Palo Alto, California, USA

**In Response**: We appreciate Dr Tang’s insightful letter (*1*) regarding our article (*2*) and are encouraged that this case report may have evoked increased interest in the phylogeny of the influenza sequences obtained during this patient’s longstanding illness. We were acutely aware that we had not provided evidence that it was indeed the same influenza A virus found in these samples. Even though we proposed sequencing studies in the article’s discussion section, in hindsight, we should have further expanded the discussion to include the points Dr Tang raises. At that time, however, we made a conscious decision not to include sequencing data in the manuscript for the following reasons.

First, we believed that finding that influenza A virus could be isolated from stool needed to be rapidly disseminated during the pandemic to reinforce awareness of the potential risk of acquiring influenza A infection through this source. During the summer of 2009, when we wrote this article, only influenza A (H5N1) had been reported to be culturable from human stool (*3*). Viral nucleic acids of seasonal influenza A had been demonstrated in stool in several studies (*4**–**6*), but of course, identification of viral nucleic acids remains an imperfect correlate to the presence of infectious virus.

Second, we considered the time, personnel, and funding required for a viral sequencing project of the type suggested and determined that we should attempt this as a follow-up study. Samples were obtained from the patient nearly every other week for >2 years, providing valuable data to further investigate this important question.

Dr Tang makes an addditional point regarding the use of ribavirin to treat the patient’s condition. As laboratorians, we had similar questions and never found satisfactory answers. The patient did eventually receive oseltamivir, but this occurred >4 months after his last positive influenza A test result. The antiviral drug course was given empirically after the patient was admitted to the pediatric intensive care unit with fever and mental status changes, which were ultimately determined to be due to coagulase-negative staphylococcal septicemia.

## References

[1] Tang JW. The persistence of influenza infection [letter]. Emerg Infect Dis. 2010;16:1817–8.2102956110.3201/eid1611.100974PMC3294535

[2] Pinsky BA, Mix S, Rowe J, Ikemoto S, Baron EJ. Long-term shedding of influenza a virus in stool of an immunocompromised child. Emerg Infect Dis. 2010;16:1165–7. 10.3201/eid1607.09124820587197PMC3321893

[3] de Jong MD, Simmons CP, Thanh TT, Hien VM, Smith GJ, Chau TN, Fatal outcome of human influenza A (H5N1) is associated with high viral load and hypercytokinemia. Nat Med. 2006;12:1203–7. 10.1038/nm147716964257PMC4333202

[4] Dilantika C, Sedyaningsih ER, Kasper MR, Agtini M, Listiyaningsih E, Uyeki TM, Influenza virus infection among pediatric patients reporting diarrhea and influenza-like illness. BMC Infect Dis. 2010;10:3. 10.1186/1471-2334-10-320053294PMC2820480

[5] Wootton SH, Scheifele DW, Mak A, Petric M, Skowronski DM. Detection of human influenza virus in the stool of children. Pediatr Infect Dis J. 2006;25:1194–5. 10.1097/01.inf.0000245097.95543.1117133173

[6] Chan MC, Lee N, Chan PK, Leung TF, Sung JJ. Fecal detection of influenza A virus in patients with concurrent respiratory and gastrointestinal symptoms. J Clin Virol. 2009;45:208–11. 10.1016/j.jcv.2009.06.01119540157
